# A Systematic Study of Dysregulated MicroRNA in Type 2 Diabetes Mellitus

**DOI:** 10.3390/ijms18030456

**Published:** 2017-02-28

**Authors:** Yuqing He, Yuanlin Ding, Biyu Liang, Juanjuan Lin, Taek-Kyun Kim, Haibing Yu, Hanwei Hang, Kai Wang

**Affiliations:** 1 Institute of Medical Systems Biology, Guangdong Medical University, Dongguan 523808, China; 2 Department of Epidemiology and Medical Statistics, Guangdong Medical University, Dongguan 523808, China; gdmcsbd@163.com (Y.D.); liangbiyu2017@hotmail.com (B.L.); linjuanjuan2017@hotmail.com (J.L.); hbyu616688@163.com (H.Y.); 3 Institute for Systems Biology, Seattle, WC 98109, USA; tkim@systemsbiology.org; 4 Department of endocrinology, Zhongshan Chen Xinghai Hospital Affiliated to Guangdong Medical University, Zhongshan 528415, China; HuangHW662@163.com

**Keywords:** microRNA, type 2 diabetes mellitus, miRNA-mRNA interaction network, systematic study

## Abstract

MicroRNAs (miRNAs) are small noncoding RNAs that modulate the cellular transcriptome at the post-transcriptional level. miRNA plays important roles in different disease manifestation, including type 2 diabetes mellitus (T2DM). Many studies have characterized the changes of miRNAs in T2DM, a complex systematic disease; however, few studies have integrated these findings and explored the functional effects of the dysregulated miRNAs identified. To investigate the involvement of miRNAs in T2DM, we obtained and analyzed all relevant studies published prior to 18 October 2016 from various literature databases. From 59 independent studies that met the inclusion criteria, we identified 158 dysregulated miRNAs in seven different major sample types. To understand the functional impact of these deregulated miRNAs, we performed targets prediction and pathway enrichment analysis. Results from our analysis suggested that the altered miRNAs are involved in the core processes associated with T2DM, such as carbohydrate and lipid metabolisms, insulin signaling pathway and the adipocytokine signaling pathway. This systematic survey of dysregulated miRNAs provides molecular insights on the effect of deregulated miRNAs in different tissues during the development of diabetes. Some of these miRNAs and their mRNA targets may have diagnostic and/or therapeutic utilities in T2DM.

## 1. Introduction

Diabetes mellitus has become a global epidemic. It affects roughly 8.3% of the population, with 90% of these patients suffering from type 2 diabetes mellitus (T2DM) [1]. T2DM is a complex disease caused by both genetic and environmental factors [2]. It often occurs in middle-aged adults with chronic hyperglycemia. Lifestyle changes are often used to prevent or manage the progression of the disease. However, the pancreatic β-cell dysfunction usually progresses and results in insulin resistance and/or relative insulin insufficiency in key metabolic organs, such as skeletal muscle, liver and adipose tissues [3]. Long-term de-regulation of energy metabolism in T2DM leads to systemic complications, such as CAD (coronary artery disease), stroke, peripheral arterial disease [4], CKD (chronic kidney disease) [5], microvascular diseases (retinopathy) [6] and various other conditions, such as cancers [7,8,9,10,11].

MicroRNAs (miRNAs) are 17–21 nt regulatory RNAs that suppress the translation and stability of mRNA through imperfect base pairing in the 3′ untranslated region of its mRNA targets [12]. There are over 2500 human miRNAs deposited in the latest release of miRBase (available online: www.mirbase.org). It has been estimated that more than 60% of protein coding transcripts are regulated by one or more miRNAs [13]. A number of dysregulated miRNAs from insulin-sensitive organs, including skeletal muscle, white adipose tissue and insulin-producing pancreatic β-cells, has been linked to processes related to diabetes, such as insulin secretion, pancreatic β-cells and adipocyte differentiation [14]. Systematic studies on the involvement of these aberrant miRNAs in T2DM will enhance our understanding of the molecular processes involved in the development of T2DM and facilitate the development of new disease management tools, therapeutic targets, diagnostic markers and preventive measures for T2DM.

Many studies have characterized the changes and involvement of miRNA in different cell types and organs during the development of T2DM [15,16,17,18,19,20,21,22]. Due to the complexity of T2DM pathology, a thorough systematic comparison, integration and investigation on the functional implications of these dysregulated miRNAs is still needed. We searched all reports related to miRNA and T2DM, downloaded and analyzed the data that met our inclusion criteria and identified a set of dysregulated miRNAs from 59 independent studies. Based on their predicted and validated targets, we conducted pathway enrichment analysis and built mRNA-miRNA interaction networks that help our understanding of the effects of these aberrantly expressed miRNAs in T2DM. The identified miRNA meta-signature and perturbed networks associated with T2DM will facilitate our understanding of the underlying biological processes in the development of T2DM.

## 2. Results

### 2.1. Study Characteristics

**Figure 1:**
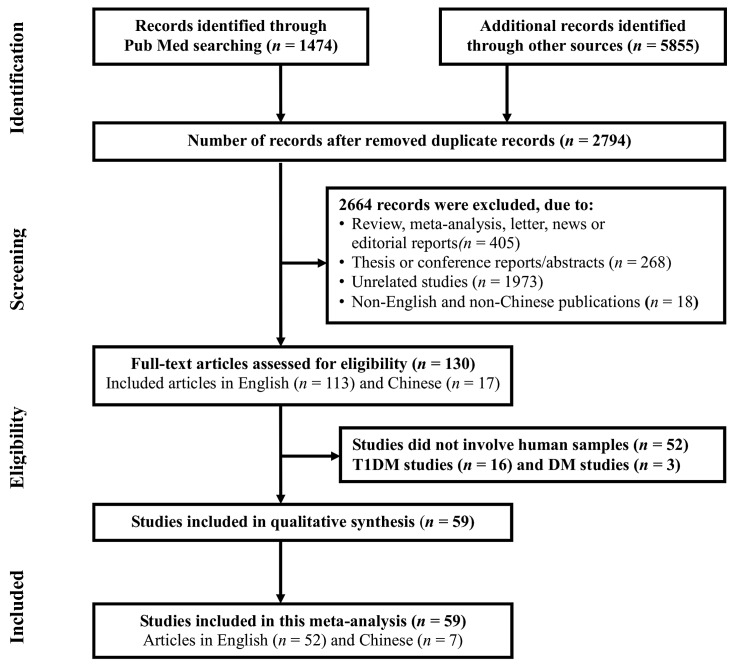
The flow chart of the data selection and identification process.

The schema of data identification and selection is shown in Figure 1. According to the search criteria outlined, a total of 7329 publications were identified through initial searches of various databases using different combinations of terms as described in the Materials and Methods section. During the data extraction step, 2794 duplicated records were removed, and an additional 2664 studies were also excluded due to either review, meta-analysis, letter, news or editorial articles (*n* = 405); thesis or conference reports/abstracts (*n* = 268); unrelated studies (*n* = 1973); non-English or Chinese publications (*n* = 18). After detailed evaluation, another 71 studies were removed due to not using human samples (*n* = 52), being T1DM studies (*n* = 16) and studies without specify T1 or T2DM (*n* = 3). At the end, the results from 59 published studies were retrieved from public databases, which include a total of 2671 T2DM patients and 2573 healthy controls (Figure 1).

The main characteristics extracted from various datasets are summarized in Table 1, which includes the number of patients investigated, the measurement platform, type of sample used and the identity of miRNAs in the study [23,24,25,26,27,28,29,30,31,32,33,34,35,36,37,38,39,40,41,42,43,44,45,46,47,48,49,50,51,52,53,54,55,56,57,58,59,60,61,62,63,64,65,66,67,68,69,70,71,72,73,74,75,76,77,78,79,80,81]. Comparing results from multiple tissues provides an overall view of the impact of miRNAs in T2DM pathology. Before analysis, we updated the miRNA name according to the latest nomenclature provided by miRBase. Three miRNAs, miR-463, miR-768 and miR-801, were removed from analysis due to these miRNAs no longer being recognized as miRNAs. Several major sample types were involved in the 59 studies, including adipose tissue, islet, skeletal muscle, whole blood, PBMC (peripheral blood mononuclear cell), serum and plasma. Some miRNAs showed inconsistent changes among different studies within the same sample type, and these miRNAs are indicated in Table 2 and Table 3. For studies that included profiling, as well as validation components, we selected validated miRNAs for further study; for example in the study by Karolina et al. [26], eight qRT-PCR validated differentially expressed miRNAs (miR-29a, -144, -150, -192, -320a, -30d, -146a and miR-182) identified by microarray profiling results were included in our analysis (Table 1).

### 2.2. Quality Assessment

The quality assessment scores listed in Table 1 were conducted in accordance with the QUADOMICS tool. None of the studies were classified as “low quality”, fulfilling fewer than 12 of the 16 criteria. Since none of the studies were conducted as blind tests (without knowledge of the reference standard and patient sample), all studies failed Criteria 12 and 13 of the QUADOMICS tool.

### 2.3. Alterations of Specific miRNAs’ Levels among Different Tissues in T2DM

From 59 independent reports, a total of 158 dysregulated miRNAs were identified (Table 1 and Table 2). According to the sample type used, results from most of the studies can be divided into seven major groups (adipose, islet, skeletal muscle, whole blood, PBMC, plasma and serum).

Skeletal muscle had the most number of affected miRNAs: 29 with decreased concentration and 31 with increased concentration in skeletal muscle samples from T2DM patients compared to healthy controls (Table 2). As expected, there are more aberrantly-expressed miRNAs in common among whole blood, PBMC, serum and plasma (Table 3). This is especially true between serum and plasma, since these are prepared from a common source. Between serum and plasma, there are 16 affected miRNAs in common; however, the concentration changes between the two may be different. For example, the levels of miR-191-5p and miR-192-5p are decreased in both serum and plasma in T2DM patients compared to healthy controls, but the concentration changes of miR-29b-3p and miR-320a are opposite between serum and plasma (Table 2 and Table 3). The concentration of miR-375, a highly enriched miRNA in pancreatic tissue, is increased in the islet of T2DM patients, as well as in whole blood, serum and plasma. There are also some common changes between skeletal muscle and whole blood, for example miR-100-5p, miR-126-3p and miR-144-3p. This suggests some of the deregulated circulating cell-free miRNAs might reflect T2DM-associated pathologies in different tissues.

### 2.4. Perturbed Pathways Mediated by Dysregulated miRNAs

Using the list of dysregulated miRNAs in each sample type identified from the literature, we performed pathway enrichment analysis based on validated and predicted miRNA targets (Table S1). Some of these pathways are known to be involved in T2DM; for example, various pathways associated with metabolic processes (carbohydrate and lipid metabolism), cell-cell communications (focal adhesion, tight junction), cell growth and death (apoptosis and cell cycle), signal transduction (JAK-STAT, MAPK, TGF-β, Wnt, cytokine-cytokine receptor interaction and neurotrophin signaling), immune response (leukocyte transendothelial migration, T-cell receptor signaling, Nod-like receptor signaling, Toll-like receptor signaling and chemokine signaling), insulin signaling and type 2 diabetes signaling.

**Figure 2:**
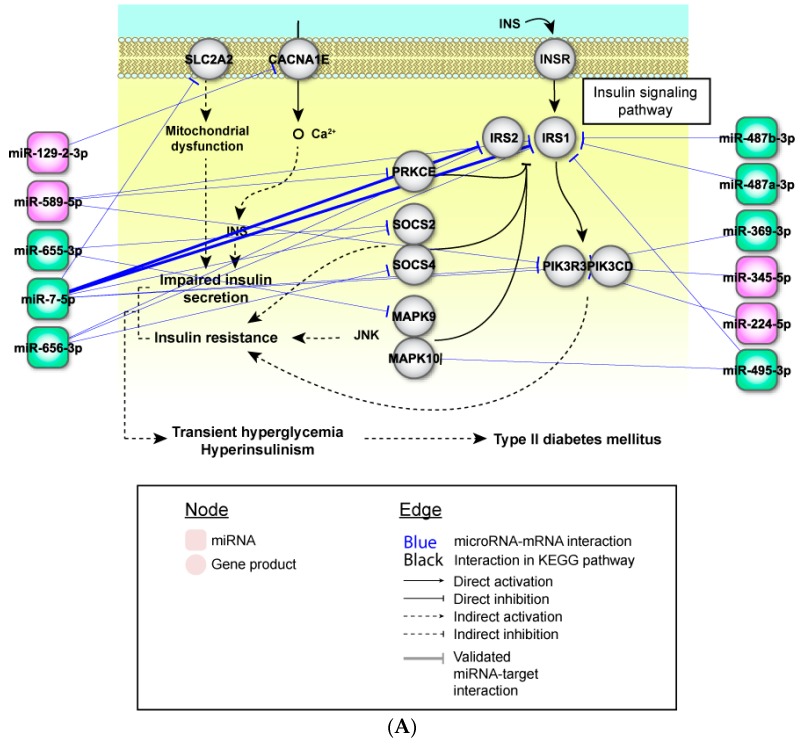

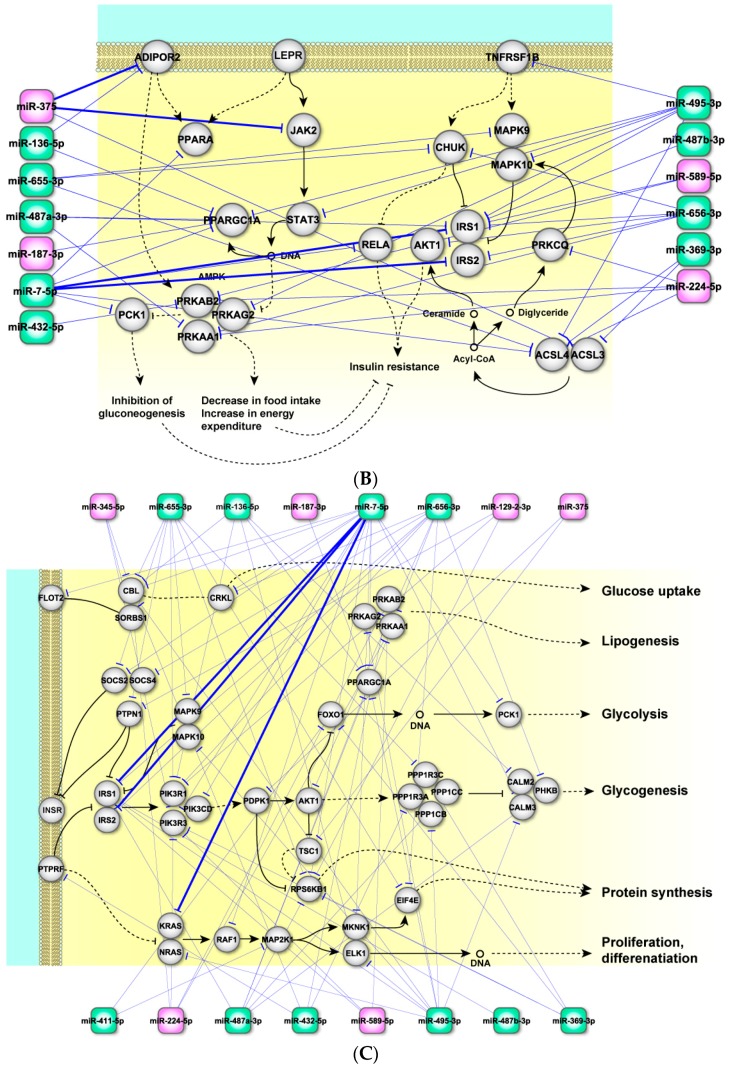
Schematic diagram of the microRNA-mRNA interaction networks in pancreatic islets. The networks are built based on the KEGG pathway map: T2DM pathway (KEGG hsa04930) (**A**); adipocytokine pathway (KEGG hsa04920) (**B**); and insulin signaling pathway (KEGG hsa04910) (**C**). The genes are indicated by circles and miRNAs by squares. For detailed descriptions of node shape, edge size, edge color and arrow shapes see inserted legend under (**A**). The predicted miRNA-mRNA interactions are indicated by light blue lines, and the thick blue lines indicate validated miRNA-mRNA interactions. Interactions denoted in KEGG pathways were presented as black solid or dotted lines indicating direct or indirect interactions, respectively. The identity of genes and miRNAs involved in the process are listed, and the colors indicate the expression level changes in T2DM islets compared to the control; red indicates a higher level in patients; and green represents lower levels compared to the control.

**Figure 3:**
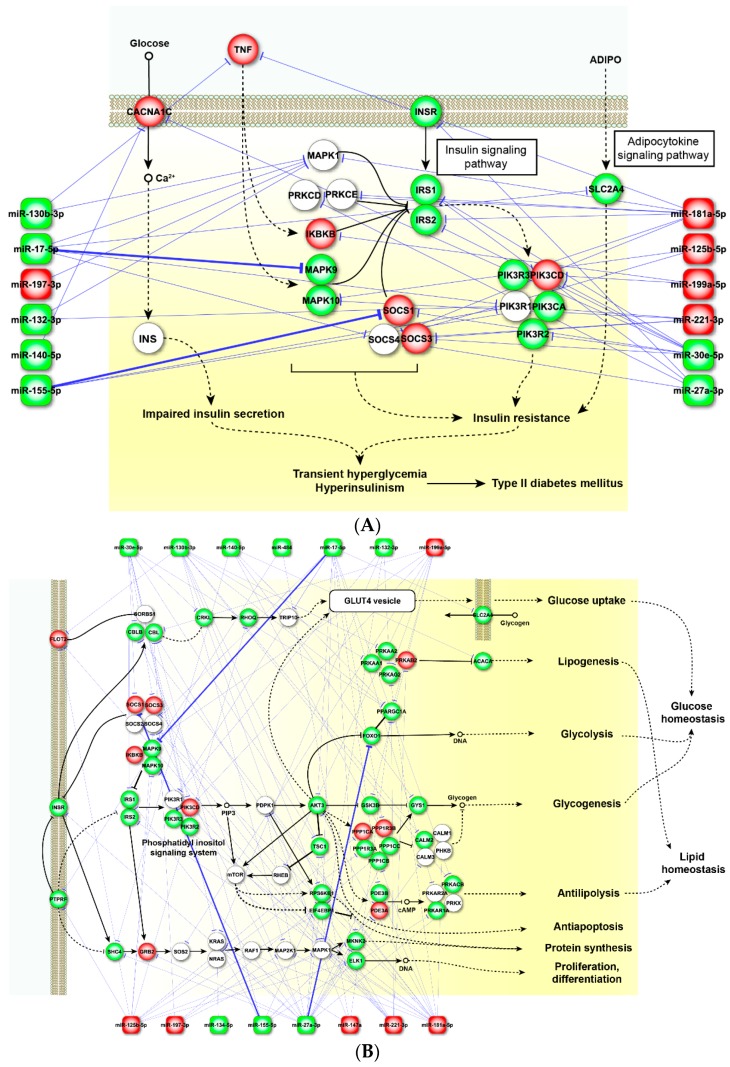

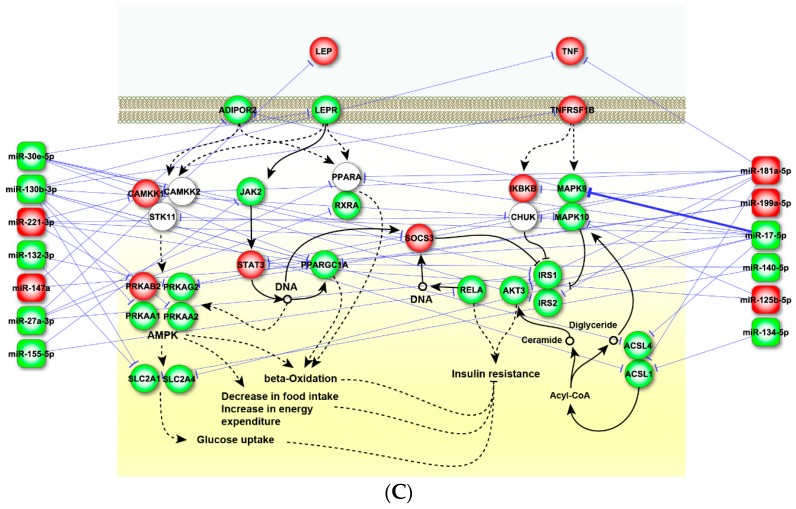
Schematic diagram of the microRNA-mRNA interaction networks in adipose tissue. The networks are built based on the KEGG pathway map: T2DM pathway (KEGG hsa04930) (**A**); adipocytokine pathway (KEGG hsa04920) (**B**); and insulin signaling pathway (KEGG hsa04910) (**C**). The genes are indicated by circles and miRNAs by squares. See the legend of Figure 2A for descriptions of node shape, edge size, edge color and arrow shapes. The identity of genes and miRNAs involved in the process are listed, and the colors indicate the relative expression changes in T2DM adipose tissues compared to the control; red indicates higher level in patients; and green represents lower levels compared to the control. The mRNA expression levels were obtained from GSE16415 (available online: https://www.ncbi.nlm.nih.gov/geo/query/acc.cgi?acc=GSE16415).

Based on the KEGG (Kyoto Encyclopedia of Genes and Genomes) pathway map and affected miRNAs from each sample type, we constructed miRNA-mRNA interaction networks for three key T2DM-associated pathways, T2DM signaling, insulin signaling and adipocytokine pathways in pancreatic islet (Figure 2) and adipose tissues (Figure 3), to better illustrate the complex interactions between mRNAs and affected miRNAs in key tissues associated with the manifestation of T2DM. Since adipose tissue is one of the key organs involved in the development of T2DM, we included the expression level changes of protein coding mRNAs involved in the pathways from a visceral adipose tissue expression profiling dataset obtained from the public domain (GSE16415, available online: https://www.ncbi.nlm.nih.gov/geo/query/acc.cgi?acc=GSE16415). The dataset compared adipose tissues gene expression differences between women with type 2 diabetes with age and BMI matched normal women. The omental biopsies samples were from five diabetic and five control females undergoing cholecystectomy. All subjects used in the study were older than 50 years with BMI values greater than 30. In both pancreatic islet and adipose tissues, the dysregulated miRNAs target several well-known T2DM-associated genes. For example, the adiponectin receptor 2 (*ADIPOR2*) in the adipocytokine pathway is targeted by miR-375 and miR-136-5p in pancreatic islet and miR-146a in adipose tissues. The key enzymes in lipid biosynthesis and fatty acid degradation, acyl-coA synthetase long-chain family members (*ACSL1*, *2*, *3* and *4*), are also targeted by multiple miRNAs, including miR-17-5p, -130b-3p, -134-5p and -181a-5p in adipose tissue and miR-7-5p, -369-3p, -495-3p and -655-3p in islets.

## 3. Discussion

Even though the etiology of T2DM is yet to be fully understood, insulin resistance and pancreatic β-cell dysfunction are the two major causes for various T2DM-associated clinical phenotypes [3], and these conditions usually occur years before the clinical diagnosis of T2DM [82]. Many studies have suggested that miRNA is one of the key players in the pathogenesis of T2DM and associated complications, such as cardiovascular conditions and kidney dysfunction [83,84,85]. The current analysis identified 158 T2DM-associated dysregulated miRNAs in seven different sample types. Pathway enrichment analysis revealed that a number of T2DM relevant pathways is targeted by these dysregulated miRNAs. For example, the disturbed insulin signaling pathway might be one of the key reasons for the glucose and lipid metabolism impairments, which cause insulin insufficiency in skeletal muscle, liver and adipose tissues [86]. The other interesting finding is the observation that some kidney and cardiovascular function-related pathways were also affected by these dysregulated miRNAs (Table S1). For example, the vasopressin-regulated water reabsorption pathway is enriched in genes that are targeted by dysregulated miRNAs in adipose tissues. The other example is the association between various cardiovascular disease processes and dysregulated miRNAs in serum.

It is well-known that hyperglycemia, hyperlipidemia and islet β cell dysfunction are associated with the onset of T2DM [82]. Our analysis also shows that a number of aberrant miRNAs are involved in pathways associated with energy metabolism, insulin signaling, type 2 diabetes signaling and adipocytokine signaling (Table S1). Among the 19 dysregulated miRNAs in pancreatic islets, miR-375 is probably the best characterized miRNA that has been shown to be involved in both insulin secretion, release and glucose homeostasis [39,87]. Increased miR-375 levels in the islets inhibit *PDPK1* (3-phosphoinositide-dependent protein kinase 1) expression [31], an important component of the PI3k/protein kinase B signal cascade (Figure 2B, insulin signaling pathway). Loss of PDPK1 in β cells results in progressive hyperglycemia due to reductions in the number and size of β cells, as well as defective β cell function [88]. In addition to miR-375, other dysregulated miRNAs in the islets (such as miR-7-5p, -369-5p, -129-3p,-136-5p, -187-3p, -589-5p, -224-5p, -655-3p, -495-3p) affect the expression of *IRS1*, *IRS2*, *AKT1*, *PPARA*, *MAPK9*, *MAPK10*, *STAT3*, *PPKAG2*, *ACSL3* and *ACSL4*, which are important genes involved in insulin signaling and type 2 diabetes pathways [89,90] (Figure 2A–C), and have been implicated in various events associated with islet β cell development and glucose-stimulated insulin secretion (GSIS) [23,36,90,91].

Adipose tissues play a key role in maintaining the homeostasis of the body’s energy metabolism by releasing an array of hormones and cytokines: adipocytokines. The current study revealed that the dysregulated miRNAs play important roles in glucose, as well as lipid metabolism (Table S1) [29,50,92]. These miRNAs, including miR-17-5p, -155-5p, -125b-5p, -30e-5p, -27a-5p, -221-3p, -199a-5p, -130b-3p, -181a-5p, -29a, -29b, interact with multiple transcription factors, such as *PPARs* (peroxisome proliferator activated receptors), including *PPARG*, also known as *PPARγ*, and adipocyte-enriched genes (*GLUT4* (also known as *SLC2A4*), *SOCS1*, *SOCS3*, *GRB2*, *INSR* and *PPARG*), to regulate many aspects of the lipid and glucose metabolisms [90,93]. Some of these miRNAs (miR-130b-3p, -140-5p, -147a, -199a-5p, -27b, -221-3p and -30e-5p) have also been implicated in the regulation of adipogenesis through *PPARG* [94,95]. miR-181a-5p, -132-3p, -199a-5p, -24-3p and -126-3p also contribute to an increased glucose uptake by affecting PI3K/AKT activity, where the PI3K/ATK signaling is essential for *SLC2A4* (*GLUT4*) translocation to the cell membrane. In addition, miR-130b-3p, -132-3p, -181a-5p, -199a-5p and -221-3p can directly target PPKAs (AMP-activated protein kinase) to affect energy metabolism in various signal transduction pathways, including adipocytokine and insulin signaling pathways [90,93] (Figure 3A–C). Dysfunction of adipose tissues may induce inflammation and insulin resistance by releasing pro-inflammatory cytokines, such as TNFA (tumor necrosis factor alpha) and IL-6 (interleukin 6), as well as energy regulating hormones including LEP (leptin), ADIPOQ (adiponectin), RETN (resistin) and ITLN1 (intelectin 1) [96]. The aberrant expression of these genes/proteins will further the manifestation of T2DM.

Skeletal muscle is one of the main tissues involved in glucose uptake and metabolism. Insulin resistance in skeletal muscle is an early sign of T2DM and is a risk factor for cardiovascular diseases common among patients with T2DM [49,86]. Studies have indicated that several miRNAs (miR-17-5p, -24, -126, -125b-5p, -130b-3p, -132-3p, -181a-3p, -181a-5p, -197-3p and -221-3p) can target and affect MAPK protein (mitogen-activated protein kinases) levels under high glucose condition [49,97,98]. MAPKs can then affect the level of MEF2 (myocyte enhancer factor 2), which is a key muscle-specific transcription factor to regulate the transcription of the insulin-responsive SLC2As (solute carrier family 2 transporters, which facilitate glucose transport), including GLUT4 (glucose transporter 4, SLC2A4) [49,93].

There are a number of studies investigating the relationship of miRNA and diabetes based on animal models and cell line systems [99,100,101,102,103]. These studies provide valuable information, but in the current analysis, we did not include these studies because the model systems, either in vivo or in vitro, can only reflect very limited aspects of the T2DM pathology. The physiopathological difference between human and model organisms will also generate a very different set of dysregulated genes and miRNAs [104,105]. Moreover, even the same miRNA may interact with very different sets of targets due to a difference in both mRNA and miRNA sequences between human and animal models. For example, miR-375 has an identical mature sequence between human (hsa-miR-375) and mouse (mmu-miR-375), but the peroxisome proliferative activated receptor, gamma, coactivator 1 alpha (*PPARGC1A*), a transcriptional coactivator, interacts with PPARgamma (*PPARG*) to regulate genes involved in energy metabolism and is a target of mouse miR-375, but not human. Therefore, in the current study, we only selected studies from human patient samples in order to investigate the functional involvement of miRNAs in human T2DM pathology. These findings may provide a more accurate and more realistic view of the effect of miRNAs in diabetes.

Some of the miRNA-mRNA interactions in Figure 2 and Figure 3 are based on results from various miRNA target prediction programs. We used miRSystem, an integrated miRNA function prediction website based on multiple miRNA target prediction algorithms, to facilitate the summarization of miRNA-mRNA interaction [106]. There are other similar websites, such as miRGator (available online: www.mirgator.kobic.re.kr/) [107] and miRPathDB (available online: mpd.bioinf.uni-sb.de/) [108]. All of these tools provide a similar function and deliver comparable results.

In the past few years, there have been a number of reports revealing disease-associated concentration changes of specific miRNA in circulation, such as miR-375 in patients with T2DM (Table 3) [31,34,39,75]. However, the idea of using circulating miRNA to reflect pathological changes in specific tissues and/or as a reliable biomarker for disease diagnosis faces numerous challenges. These difficulties are due to many issues, including the accuracy and consistency of different miRNA measurement platforms, low concentration of circulating RNA in samples and the high sequence similarity of miRNA family members. However, the biggest challenge of circulating RNA-based biomarker discovery probably is due to the fact that the spectrum of circulating RNA is the sum of many different tissues and cell types in the body. How to identify changes from specific tissues or disease stages from very complex and noisy data is difficult. Using tissue or disease-enriched RNAs (miRNAs or mRNAs), like miR-375, which is enriched in pancreatic tissue, may provide a useful approach for circulating RNA-based biomarker discovery. In addition, using animal models that allow frequent and detailed monitoring of disease-associated RNA profile changes in tissue and body fluid samples may also provide insights on the relationship of RNA between the two compartments.

In conclusion, we present a systematic analysis by integrating various T2DM-related miRNA datasets from the literature. These dysregulated miRNAs affect various metabolic and signal transduction pathways associated with T2DM and may play important roles in the development of diabetes. The pathway analysis results also provide possible links to kidney and cardiovascular complications associated the T2DM. However, many other factors, both endogenous and exogenous, may influence the spectrum of miRNA in tissues and circulation. Recent findings suggest that diet, a key component in T2DM disease management, affects the spectra of miRNA in various tissues [109,110]. For example, the consumption of milk resulted in significant increases of miRNA levels in plasma and PBMCs [111]. These findings suggested that individual’s diet and nutritional status can influence miRNA in tissues and circulation. Therefore, more studies with larger and well-defined populations are clearly needed to resolve this complex miRNA-mediated regulatory network. The dysregulated miRNAs and their interacting mRNA targets may provide new insights to the T2DM pathology and provide new disease monitoring and management tools.

## 4. Materials and Methods

### 4.1. Search Strategy and Eligibility of Relevant Studies

We scanned all relevant studies published prior to 18 October 2016 through a comprehensive search of records in PubMed, The Cochrane Library, the Chinese National Knowledge Infrastructure (CNKI) database (available online: http://www.cnki.net), Wanfang database (available online: http://www.wanfangdata.com), VIP database (VIP information/Chinese Scientific Journals database, available online: http://www.cqvip.com) and Google Scholar. The search was conducted by using combinations of the following key words: “miRNA” or “microRNA” and “diabetes” or “type 2 diabetes”. The online search was accompanied by checking cited references from the articles for potentially eligible reports. References of identified studies that met the inclusion criteria have been assessed by two independent reviewers. To ensure that relevant studies were not missed, searches in Gene Expression Omnibus (GEO, available online: www.ncbi.nlm.nih.gov/geo/) and Array Express (available online: www.ebi.ac.uk/arrayexpress) repositories were also performed.

### 4.2. Inclusion and Exclusion Criteria

All miRNA studies associated with T2DM were included in the current meta-analysis if they met all of the following criteria: (1) full text of original experimental articles based on miRNA expression profiling between diabetic patients and normal individuals; (2) studies based on human samples; and (3) original articles published in a language of either English or Chinese. The main exclusion criteria were as follows: (1) meta-analyses, series, abstract, commentary, review, letters and editorial; (2) duplicate data; (3) studies that compared different stages of diabetes, but did not include normal samples; and (4) studies based on cell lines or animal models.

### 4.3. Data Extraction

Data were extracted by two independent reviewers from all publications that met the inclusion criteria. The lists of miRNAs with statistically-significant expression changes were extracted from the publications. All miRNA names were standardized according to miRBase Version 21 (Table 1 and Table 2). Any discrepancies were resolved by consensus or in consultation with a third reviewer. Information extracted from eligible publications includes: first author’s last name, year of publication, country of origin, population ethnicity, disease type, measurement platform, source of cases and control groups, number of samples and number of miRNAs measured. All relevant information was compiled in Table 1.

### 4.4. Quality Assessment and Statistical Analysis

We assessed the quality of the data using the QUADAS2 (Quality Assessment of Diagnostic Accuracy Studies 2) checklist. The categories in the scoring system used for assessing study quality are summarized in Table 1. Quality scores ranged from 0–10, and studies were scored as “good” if the score was 8–10, “fair” if the score was 5–7 and “poor” if the score was <4. The dysregulated miRNAs were grouped into seven groups based on the different sample sources used in the studies (adipose, islet, skeletal muscle, whole blood, PBMC, plasma, serum).

### 4.5. miRNA Target Prediction and Pathway Enrichment Analysis

To identify the functional involvement of miRNAs in the progression of T2DM, we used miRNA target prediction algorithms and information from validated miRNA target databases to generate the list of putative miRNA targets. The list of putative interacting targets for the miRNAs was generated using the miRSystem (available online: http://mirsystem.cgm.ntu.edu.tw/index.php) web server, which provides information from seven miRNA target prediction algorithms, DIANA-microT, miRanda, mirBridge, PicTar, PITA, RNA22 and TargetScan, and two experimentally-validated miRNA target databases, TarBase and miRecords [106]. To increase the reliability of target prediction, the targets that have been predicted by more than 5 different algorithms were then used for pathway enrichment analysis (available online: http://david.abcc.ncifcrf.gov/home.jsp). Cytoscape (available online: http://www.cytoscape.org/) and KEGG pathway maps (available online: http://www.genome.jp/kegg/pathway.html) were used to generate miRNA-mRNA interaction networks. We also included the mRNA expression level changes from visceral adipose tissue expression profiling data from the public domain (GSE16415).

## Figures and Tables

**Table 1:** Main characteristics of the reports included in the study.

Author, Year (Ref.)	Country	Sample Type	miRNA	Exp Change	Assay Method	Number of Samples	Avg Age	Gender (M/F)	QC
Ding et al., 2016 [63]	China	Serum	miR-451a, -4534	Up	RT-qPCR	T2DM(40)/NC(56)	61.21	59/37	9
			miR-320d, -3960, -572	Down					
Hou et al., 2016 [62]	China	Pancreatic islets	miR-463-3p	Up	RT-qPCR	T2DM(34)/NC(34)	53 ± 7.5	35/33	7
Jansen et al., 2016 [61]	Germany	Plasma	miR-126, -26a	Down	RT-qPCR	T2DM(55)/NC(80)	66.4 ± 10.9	45/90	7
Latouche et al., 2016 [60]	Australia	SM	miR-194	Down	RT-qPCR	T2DM(6)/NC(5)	50.72	NA	8
Li et al., 2016 [59]	China	Serum	miR-221/222	Up	RT-qPCR	T2DM(30)/NC(20)	60.28	NA	7
Pek et al., 2016 [58]	China, Malaysia, India and Others	Adipose tissue	miR-100, miR-378, miR-99a, miR-125b, miR-181a, miR-210 and miR-378	Down	Microarray RT-qPCR	T2DM(42)/NC(73)	40.4 ± 11.25	NA	7
Rezk et al., 2016 [57]	Egypt	Serum	miR-126	Down	RT-qPCR	T2DM(100)/NC(100)	46.95	95/105	9
Seyhan et al., 2016 [56]	USA	Plasma	miR-30d, -34a, -21, -148a	Up	RT-qPCR	T2DM(31)/NC(27)	40.05	30/28	9
Yan et al., 2016 [53]	China	Plasma	miR-572	Up	Microarray RT-qPCR	T2DM(50)/NC(50)	45.87	49/51	8
			miR-1249, -320b	Down					
Wang et al., 2016 [55]	China	Serum	miR-661, -571, -770-5p, -892b, -1303, -15a, -16, -125b, -221, -320a	Up	RT-qPCR	T2DM(92)/NC(92)	48.95	114/70	8
Baldeon et al., 2016 [64]	Ecuador	Serum	miR-574-3p, -146a	Down	RT-PCR	T2DM(64)/NC(44)	61 (37–85)	37/71	8
Wang et al., 2016 [54]	China	Plasma	miR-296, -9	Down	RT-qPCR	T2DM(150)/NC(150)	48.6 ± 1.7	150/150	7
Long et al., 2015 [67]	China	PBMC	miR-223-3p	Down	RT-qPCR	T2DM(16)/NC(18)	55	20/14	6
Olivieri et al., 2015 [66]	Italy	PBMC	miR-126-3p, -21-5p	Down	RT-qPCR	T2DM(76)/NC(107)	64.79	85/98	9
Yang et al., 2015 [52]	China	Plasma/platelets	miR-144	Up	RT-qPCR	T2DM(114)/NC(30)	49.8 ± 9.1	NA	8
			miR-223	Down					
Higuchi et al., 2015 [70]	Japan	Serum	miR-101, -375, -802	Up	RT-qPCR	T2DM(155)/NC(49)	62.3 ± 13.2	121/83	7
Al-Kafaji et al., 2015 [73]	Bahrain	WB	miRNA-15a	Down	RT-qPCR	T2DM(24)/NC(24)	52 ± 6.0	23/25	8
Lenin et al., 2015 [68]	India	PBMC	miR-146a	Down	RT-qPCR	T2DM(35)/NC(35)	47.3 ± 7	36/34	6
Sebastiani et al., 2015 [65]	Italia	Pancreatic islets	miR-124a	Up	RT-qPCR	T2DM(5)/NC(10)	71.2 ± 9.8	7/8	7
Jiao et al., 2015 [69]	China	PB	miR-130a, -10b, -143	Down	RT-qPCR	T2DM(30)/NC(42)	56 ± 10	NA	8
Bao et al., 2015 [71]	China	Plasma/Serum	miR-185	Down	RT-qPCR	T2DM(34)/NC(30)	NA	NA	9
Baldeón et al., 2015 [72]	Ecuador	PBMC	miR-34c-5p, -576-3p	Up	Microarray RT-qPCR	T2DM(64)/NC(44)	61 (37–85)	37/71	6
Wu et al., 2015 [79]	China	PBMC	miR-21	Up	RT-qPCR	T2DM(18)/NC(18)	53.6 ± 4.6	18/18	7
Ortega et al., 2014 [77]	Spain	Plasma	miR-140-5p, -142-3p, -222	Up	RT-qPCR	T2DM(48)/NC(45)	54 ± 10	93/0	8
			miR-423-5p, -125b, -192, -195, -130b, -532-5p, -126	Down					
Yan et al., 2014 [80]	China	Plasma	miR-199a	Up	RT-PCR	T2DM(64)/NC(64)	46–62	NA	8
Lu et al., 2014 [76]	China	Plasma	miR-375, miR-126	Up	RT-qPCR	T2DM(30)/NC(30)	53.67 ± 8.92	42/18	8
Wang et al., 2014 [78]	Swedes Iraqis	Plasma	miR-15a, -21, -144, -150, -486-5p	Up	RT-qPCR	T2DM(33)/NC(119)	45-65	83/69	7
			miR-24, -29b, -126, -320a	Down					
Liu et al., 2014 [35]	China	Serum	miR-126	Down	qPCR	T2DM(160)/NC(138)	50.2 ± 6.7	78/82	9
Pan et al., 2014 [51]	China	WB	miR-146a, -155	Down	FQ-PCR	T2DM(36)/NC(32)	61.0 ± 7.0	20/16	9
Locke et al., 2014 [36]	UK	Islet	miR-129-3p, -187, -345	Up	RT-qPCR	T2DM(9)/NC(11)	53	7/2	5
Yang et al., 2014 [40]	China	Serum	miR-23a, let-7i, -486, -96, -186, -191, -192, -146a	Down	RT-qPCR	T2DM(24)/NC(20)	50.60 ± 5.128	16/8	9
Santovito et al., 2014 [38]	Germany	Plasma	miR-326	Up	RT-qPCR	T2DM(18)/NC(12)	57.2 ± 9.6	12/6	7
			miR-let-7a,let-7f	Down					
Mao et al., 2014 [35]	China	Serum	miR-18a	Down	qPCR	T2DM(33)/NC(33)	53.8 (35–72)	13/20	6
Baldeon et al., 2014 [32]	Netherlands	Serum	miR-146a	Down	RT-qPCR	T2DM(56)/NC(40)	62 (38–85)	22/34	8
Sun et al., 2014 [39]	China	Plasma	miR-375	Up	qPCR	T2DM(100)/NC(100)	51.33 ± 11.75	54/46	9
Kameswaran et al., 2014 [23]	USA	Islet	miR-7, -136, -369, -369-3p, -411, -432, -487a, -487b, -495, -539-3p, -655, -656	Down	RT-qPCR	T2DM(20)/NC(29)	51.35 (22–65)	14/6	8
			miR-187, -187 *, -224, -589	Up					
Zhang et al., 2014 [41]	China	Serum	miR-29b	Up	RT-PCR	T2DM(50)/NC(50)	35–70	30/20	8
Ren et al., 2014 [37]	China	Plasma	miR-126	Down	RT-PCR	T2DM(40)/NC(40)	43.0 ± 11.0	24/16	9
Zhou et al., 2013 [47]	China	WB	let-7a	Up	RT-PCR	T2DM(104)/NC(62)	52.8 ± 10.4	59/45	9
Pescador et al., 2013 [45]	Spain	Serum	miR-503	Down	RT-qPCR	T2DM(13)/NC(20)	69.40 ± 7.12	7/6	8
Zhang et al., 2013 [47]	China	Plasma	miR-126	Down	RT-qPCR	T2DM(30)/NC(30)	63 ± 8.56 (42–73)	16/14	8
Agarwal et al., 2013 [42]	India	SM	miR-135a	Up	RT-PCR	T2DM(3)/NC(3)	65–75	3/0	5
Rong et al., 2013 [46]	China	Plasma	miR-146a	Up	qPCR	T2DM(90)/NC(90)	48.50 (42–56)	47/43	9
Corral, et al., 2013 [43]	México	PBMC	miR-146a, -155	Down	RT-PCR	T2DM(20)/NC(20)	46.2 (35–59)	11/9	6
Liang et al, 2013 [75]	China	Serum	miR-29a, -375	Up	RT-FQ-PCR	T2DM(48)/NC(38)	54.9 ± 9.8 (35–72)	27/21	9
Zhou et al., 2012 [81]	China	Serum	miR-181a	Up	RT-PCR	T2DM(20)/NC(20)	NA	NA	7
Meng et al., 2012 [28]	China	PBMC	miR-21, -27a, -27b, -126, -130a	Down	Microarray RT-qPCR	T2DM(15)/NC(15)	67 ± 8	7/8	8
Karolina et al., 2012 [27]	Singapore	WB	miR-17, -92a, -130a, -195, -197, -509-5p, -652	Down	Microarray RT-qPCR	T2DM(50)/NC(46)	42.02	NA	9
			miR-27a, -150, -192, -320a, -375	Up					
Balasubramanyam et al., 2011 [24]	India	PBMC	miR-146a	Down	RT-qPCR	T2DM(20)/NC(20)	43.7 ± 5.1	NA	8
Caporali et al., 2011 [25]	U.K.	Plasma	miR-503	Up	RT-PCR	T2DM(10)/NC(11)	68.09 ± 9.06	9/1	7
		SM	miR-503						
Karolina et al., 2011 [26]	Singapore	WB	miR-15a, -17, -17 *, -23a, -23b, -26a, -26b, -27a, -29b, -29c, -99b *, -106b, -125a-5p, -125b, -126, -130a, -130b, -142-3p, -151-3p, -151-5p, -183, -185, -190, -193a-3p, -194, -221, -222, -299-3p, -320b, -320c, -320d, -335, -361-3p, -375, -502-3p, -550, -550 *, -589, -620, -629, -665, -886-5p, -1285, -1301	Up	Microarray	T2DM(21)/NC(15)	43.2 (21–70)	21/0	9
			miR-7, -19a, -20a, -20b, -30c, -30e, -34b, -106a, -129-5p, -146b-5p, -185 *, -186, -340, -342-3p, -362-5p, -374b, -519e, -532-3p, -636, -637, -652, -660, -923, -1184, -1297, let-7b *, let-7d, let-7e, let-7g, let-7i	Down					
			miR-29a, -144, -150, -192, -320a	Up	RT-qPCR				
			miR-30d, -146a, -182	Down					
Kong et al., 2011 [34]	China	Serum	miR-9, -29a, -30d, -34a, -124a, -146a, -375	Up	RT-qPCR	T2DM(18)/NC(19)	47.33 ± 2.617	9/9	9
Zhao et al., 2010 [31]	Hong Kong	Pancreas	miR-375	Up	RT-qPCR	T2DM(40)/NC(15)	69 ± 13	17/23	8
Zampetaki et al., 2010 [30]	U.K.	Plasma	miR-15a, -20b, -21, -24, -29b, -126, -150, -191, -197, -223, -320, -486	Down	Microarray/RT-qPCR	T2DM(80)/NC(80)	66.3 ± 8.9	30/50	5
			miR-28-3p	Up					
Gallagher et al., 2010 [49]	U.K.	SM	miR-15b, -30b *, -30c-2 *, -32 *, -93, -106b, -138-1 *, -142-3p, -142-5p, -143, -144, -181a-2 *, -185, -193a-5p, -371-5p, -451, -503, -518c *, -589, -597, -600, -634, -658, -665, -668, -765, -921, -923, -937	Up	Microarray	T2DM(45)/NC(47)	54.8 ± 10.2	NA	7
			miR-10a, -10b, -15a, -27b, -30e, -95, -100, -126 *, -128, -133a, -152, -154, -190, -196a, -199a-3p, -199b-5p, -206, -208a, -331-3p, -342-3p, -362-3p, -374a, -374b, -378 *, -422a, -423-3p, -424, -455-5p, -519d, -768-3p, -768-5p, -801	Down					
Ortega et al., 2010 [29]	Spain	Adipose	miR-125b, -199a-5p, -221, -1229	Up	RT-PCR	T2DM(9)/NC(6)	45 ± 10	0/9	8
			miR-30a *, -130b, -484	Down					
Kong et al., 2010 [74]	China	Serum	miR-34a	Up	RT-qPCR	T2DM(18)/NC(26)	47.33 ± 2.62	23/21	
Klöting et al., 2009 [50]	Germany	Adipose	miR-147, -181a, -197	Up	Microarray	T2DM(6)/NC(9)	67 ± 2.8	NA	8
			miR-17-5p, -27a, -30e, -132, -134, -140, -155, -210	Down					
Granjon et al., 2009 [33]	France	SM	miR-1, -133a	Down	RT-qPCR	T2DM(5)/NC(15)	51 ± 2	2/3	7

Abbreviations: T2DM: type 2 diabetes; NC: normal control; NA: not available; PBMC, peripheral blood mononuclear cell; WB: whole blood; SM: skeletal muscle.

**Table 2:** List of dysregulated miRNAs identified from seven different types of samples used in T2DM studies.

**Increased in T2DM Patient ^a^**							
							miR-101-3p
							miR-124-3p
							miR-125b-5p
							miR-1303
						miR-126-3p	miR-146a-5p
						miR-140-5p	miR-15a-5p
						miR-142-3p	miR-16-5p
						miR-144-3p	miR-181a-5p
		miR-106b-5p				miR-146a-5p	miR-221-3p
		miR-135a-5p	miR-451a			miR-148a-3p	miR-222-3p
		miR-138-1-3p	miR-503-5p			miR-15a-5p	miR-29a-3p
		miR-142-3p	miR-518c-5p			miR-150-5p	miR-29b-3p
		miR-142-5p	miR-589-5p			miR-199a-5p	miR-30d-5p
		miR-143-3p	miR-597-5p			miR-21-5p	miR-320a
		miR-144-3p	miR-600			miR-222-3p	miR-34a-5p
	miR-124-3p	miR-15b-5p	miR-634	let-7a-5p		miR-28-3p	miR-375
miR-1229-3p	miR-129-3p	miR-181a-2-3p	miR-658	miR-144-3p		miR-30d-5p	miR-451a
miR-125b-5p	miR-187-3p	miR-185-5p	miR-665	miR-150-5p		miR-326	miR-4534
miR-147a	miR-187-5p	miR-193a-5p	miR-668-3p	miR-192-5p		miR-34a-5p	miR-571
miR-181a-5p	miR-224-5p	miR-30b-3p	miR-765	miR-27a-3p		miR-375	miR-661
miR-197-3p	miR-345-5p	miR-30c-2-3p	miR-921	miR-29a-3p	miR-21-5p	miR-486-5p	miR-770-5p
miR-199a-5p	miR-375	miR-32-3p	miR-93-5p	miR-320a	miR-34c-5p	miR-503-5p	miR-892b
miR-221-3p	miR-589-5p	miR-371a-5p	miR-937-3p	miR-375	miR-576-3p	miR-572	miR-9-5p
**Adipose ^b^**	**Islet ^b^**	**Skeletal Muscle ^b^**		**Whole Blood ^b^**	**PBMC ^b^**	**Plasma ^b^**	**Serum ^b^**
**T2DM(72)/NC(103)**	**T2DM(88)/NC(113)**	**T2DM(114)/NC(128)**		**T2DM(378)/NC(312)**	**T2DM(264)/NC(277)**	**T2DM(1058)/NC(1234)**	**T2DM(961)/NC(821)**
miR-100-5p	miR-136-5p	miR-100-5p	miR-208a-3p	miR-10b-5p	miR-126-3p	let-7a-5p	let-7i-5p
miR-125b-5p	miR-369-3p	miR-10a-5p	miR-27b-3p	miR-130a-3p	miR-130a-3p	let-7f-5p	miR-126-3p
miR-130b-3p	miR-411-5p	miR-10b-5p	miR-30e-5p	miR-143-3p	miR-146a-5p	miR-1249-3p	miR-146a-5p
miR-132-3p	miR-432-5p	miR-126-5p	miR-331-3p	miR-146a-5p	miR-155-5p	miR-125b-5p	miR-186-5p
miR-134-5p	miR-487a-3p	miR-128-3p	miR-342-3p	miR-155-5p	miR-21-5p	miR-126-3p	miR-18a-5p
miR-140-5p	miR-487b-3p	miR-133a-3p	miR-362-3p	miR-15a-5p	miR-223-3p	miR-130b-3p	miR-191-5p
miR-155-5p	miR-495-3p	miR-1-3p	miR-374a-5p	miR-17-5p	miR-27a-3p	miR-15a-5p	miR-192-5p
miR-17-5p	miR-539-3p	miR-152-3p	miR-374b-5p	miR-182-5p	miR-27b-3p	miR-150-5p	miR-23a-3p
miR-181a-5p	miR-655-3p	miR-154-5p	miR-378a-5p	miR-195-5p		miR-191-5p	miR-320d
miR-210-3p	miR-656-3p	miR-15a-5p	miR-422a	miR-197-3p		miR-192-5p	miR-3960
miR-27a-3p	miR-7-5p	miR-190a-5p	miR-423-3p	miR-30d-5p		miR-195-5p	miR-486-5p
miR-30a-5p		miR-194-5p	miR-424-5p	miR-509-5p		miR-197-3p	miR-503-5p
miR-30e-5p		miR-196a-5p	miR-455-5p	miR-652-3p		miR-20b-5p	miR-572
miR-378a-3p		miR-199a-3p	miR-519d-3p	miR-92a-3p		miR-21-5p	miR-574-3p
miR-484		miR-199b-5p	miR-95-3p			miR-223-3p	miR-96-5p
miR-99a-5p		miR-206				miR-24-3p	
						miR-26a-5p	
						miR-296-5p	
						miR-29b-3p	
						miR-320a	
						miR-320b	
						miR-423-5p	
						miR-486-5p	
						miR-532-5p	
						miR-9-5p	
**Decreased in T2DM patient ^a^**							

^a^ miRNAs reported in multiple studies are listed in italic boldface characters, and inconsistent concentration changes between studies from the same sample type are underlined; ^b^ the number of samples involved in patients (T2DM) and controls (NC) is indicated in parentheses under each sample type.

**Table 3:** Common changes of miRNAs in different sample types.

miRNA ID	Plasma	Serum	PBMC	Whole Blood	Skeletal Muscle	Islet	Adipose
let-7a-5p	Down			Up			
miR-100-5p				Down	Down		
miR-124-3p		Up				Up	
miR-125b-5p	Down	Up					Up
							Down
miR-126-3p	Up	Down	Down		Down		
	Down						
miR-126-5p					Down		
miR-130a-3p			Down	Down			
miR-140-5p	Up						Down
miR-142-3p	Up				Up		
miR-143-3p				Down	UP		
miR-144-3p	Up			Up	Up		
miR-146a-5p	Up	Down	Down	Down			
		Up					
miR-150-5p	Up			Up			
	Down						
miR-155-5p			Down	Down			
miR-15a-5p	Up	Up		Down	Down		
	Down						
miR-17-5p				Down			Down
miR-181a-5p		Up					Up
							Down
miR-191-5p	Down	Down					
miR-192-5p	Down	Down		Up			
miR-195-5p	Down			Down			
miR-197-3p	Down			Down			Up
miR-199a-5p	Up						Up
miR-21-5p	Up		Up				
	Down		Down				
miR-221-3p		UP					Up
miR-222-3p	Up	Up					
miR-223-3p	Down		Down				
miR-27a-3p			Down	Up			
miR-27b-3p			Down		Down		
miR-29a-3p		Up		Up			
miR-29b-3p	Down	Up					
miR-30d-5p	Up	Up		Down			
miR-30e-5p					Down		Down
miR-320a	Down	Up		Up			
miR-34a-5p	Up	Up					
miR-375	Up	Up		Up		Up	
miR-451a		Up			Up		
miR-486-5p	Up	Down					
	Down						
miR-503-5p	Up	Down			Up		
miR-572	Up	Down					
miR-589-5p					Up	Up	
miR-9-5p	Down	Up					

## Supplementary Materials

Supplementary materials can be found at www.mdpi.com/1422-0067/18/2/456/s1.

## Abbreviations

GSIS	glucose-stimulated insulin secretion
PDPK1	3-phosphoinositide dependent protein kinase 1
PPARG	peroxisome proliferator-activated receptor gamma
MAPK	mitogen-activated protein kinase
ADIPOR2	adiponectin receptor 2
ACSL	acyl-coA synthetase long-chain family members
TNFA	tumor necrosis factor alpha
SLC2As	solute carrier family 2, which facilitates glucose transport
PPKAs	AMP-activated protein kinase
MEF2	myocyte enhancer factor 2
T2DM	type 2 diabetes mellitus
miRNA	microRNA
