# Supplementary material for: A Systematic Study of Dysregulated MicroRNA in Type 2 Diabetes Mellitus
Source: Int J Mol Sci. 2017 Feb 28;18(3):456. doi: 10.3390/ijms18030456 (PMC5372489; doi:10.3390/ijms18030456)
Supplement: Supplementary file 1 [file ijms-18-00456-s001.pdf]

Table S1. Enriched pathways based on predicted and validated targets of dysregulated miRNAs identified in T2DM patients.

Pathway Description		Suppressed Pathway (increased miRNA concentration)							Enhanced Pathway (decreased miRNA concentration)							
		Adipose	Islet	Muscle	Blood	PBMC	Plasma	Serum	Adipose	Islet	Muscle	Blood	PBMC	Plasma	Serum
Carbohydrate metabolism	Amino sugar and nucleotide sugar metabolism												2.08E-02		
	Citrate cycle (TCA cycle)							8.59E-02					5.52E-02		
	Fructose and mannose metabolism							9.83E-02					6.40E-02		
	Galactose metabolism							6.56E-02							
	Glycolysis gluconeogenesis				8.77E-02			2.18E-02							
	Pentose and glucuronate interconversions	1.23E-02													
	Pentose phosphate pathway							6.56E-02							
	Propanoate metabolism		5.76E-02					6.98E-02							
	Pyruvate metabolism		7.26E-02		4.24E-02			2.94E-02							1.14E-02
	Starch and sucrose metabolism	3.22E-02													
Xenobiotics metabolism	Metabolism of xenobiotics by cyp450	5.26E-02													
Energy metabolism	Oxidative phosphorylation				7.63E-02										1.44E-02
Lipid metabolism	Sphingolipid metabolism									8.78E-02	8.78E-02				
	Steroid hormone biosynthesis	3.53E-03													
Amino acid metabolism	Arginine and proline metabolism		9.34E-02												
	Cysteine and methionine metabolism		6.44E-02					1.42E-02							
	Lysine degradation							3.47E-02							
Glycan biosynthesis and metabolism	Glycosylphosphatidylinositol (gpi)-anchor biosynthesis		4.56E-02												
	N-glycan biosynthesis	2.81E-02											3.51E-03		
	O-glycan biosynthesis									6.75E-02	6.75E-02				
Transcription	Basal transcription factors			2.64E-02				2.01E-02			7.78E-02		9.82E-03		
Folding, sorting and degradation	Protein processing in ER	3.03E-04						1.85E-02	3.69E-02				1.31E-03		
	Ubiquitin mediated proteolysis						2.95E-02	9.07E-02				4.49E-02	7.06E-03		
Signal transduction	ERBB signaling pathway							1.44E-02							
	Erbb signaling pathway	7.25E-02													
	JAK-stat signaling pathway						4.04E-02	5.33E-02					9.47E-02		
	MAPK signaling pathway		1.60E-03		5.39E-02		1.01E-02	1.37E-02	9.95E-02				1.82E-02		
	TGF-beta signaling pathway	1.04E-02			4.99E-03			4.45E-02					1.09E-04		
	Wnt signaling pathway	4.19E-02											8.87E-02		
Signaling molecules and interaction	Cytokine-cytokine receptor interaction						2.87E-03						7.50E-03	8.32E-02	
	ECM-receptor interaction							9.88E-05						5.59E-02	
	Neuroactive ligand-receptor interaction				1.33E-02						3.40E-02	5.73E-02			3.07E-02
Transport and catabolism	Peroxisome									1.47E-02		5.45E-02			
	Regulation of autophagy									7.57E-02					
Cell growth and death	Apoptosis						6.83E-02								4.82E-02
	Cell cycle												5.79E-02		7.59E-02
	P53 signaling pathway				9.36E-02		4.51E-02								2.89E-03
Cell communication	Adherens junction												5.65E-02		
	Focal adhesion							4.18E-07					7.54E-02	5.80E-02	
	Gap junction							8.64E-04	5.97E-02				8.52E-02		
	Tight junction				7.63E-02			2.05E-02							1.44E-02

Table S1. *Cont.*

Pathway Description		Suppressed Pathway (increased miRNA concentration)							Enhanced Pathway (decreased miRNA concentration)						
		Adipose	Islet	Muscle	Blood	PBMC	Plasma	Serum	Adipose	Islet	Muscle	Blood	PBMC	Plasma	Serum
Immune system	Antigen processing and presentation	5.87E-02						3.40E-02							
	B cell receptor signaling pathway	5.74E-02													
	Chemokine signaling pathway						1.50E-02	8.78E-02	1.07E-02				2.70E-02		
	Complement and coagulation cascades											6.25E-02		3.20E-02	
	Fc epsilon ri signaling pathway												6.64E-02	4.03E-02	4.03E-02
	Leukocyte transendothelial migration							6.51E-02							
	Nod-like receptor signaling pathway	4.21E-02													
	T cell receptor signaling pathway	1.96E-02						8.09E-02					4.08E-02		
Endocrine system	Adipocytokine signaling pathway		9.00E-02												
	Insulin signaling pathway											4.63E-02			1.81E-02
	PPAR signaling pathway			5.14E-02									5.17E-02		
Circulatory system	Vascular smooth muscle contraction				6.97E-02				9.91E-02				1.95E-02		
Digestive system	Pancreatic secretion						8.12E-02	7.29E-02	7.37E-02			8.23E-02			
	Protein digestion and absorption							1.23E-06							
Excretory system	Aldosterone-regulated sodium reabsorption									9.18E-02					
	Vasopressin-regulated water reabsorption	1.80E-03							1.75E-02				9.45E-02		
Cancers	Pathways in cancer						5.91E-02	3.26E-03				6.03E-02	7.29E-02		
Cardiovascular diseases	Arrhythmogenic right ventricular cardiomyopathy						3.16E-02								
	Dilated cardiomyopathy							5.30E-02					8.52E-02		
	Hypertrophic cardiomyopathy							4.87E-02							
Endocrine and metabolic diseases	Maturity onset diabetes of the young									5.69E-02					9.18E-02
	Type I diabetes mellitus							3.29E-02					9.15E-02		
	Type II diabetes mellitus												9.15E-02		
